# Supplementary material for: Revisiting evolutionary trajectories and the organization of the Pleolipoviridae family
Source: PLoS Genet. 2023 Oct 13;19(10):e1010998. doi: 10.1371/journal.pgen.1010998 (PMC10599561; doi:10.1371/journal.pgen.1010998)
Supplement: S1 Text — (DOC) [file pgen.1010998.s001.doc]

**Supplementary Results and Discussion**

***Pleolipovirus-like integrated genome***

Notably, all but one of the retrieved sequences were relatively short (> 20kb) and part of the non-binned fraction of each metagenome. Contamination assessment with CheckV based on the presence of host genes, showed that only 27 contigs (~ 10 % of the total) corresponded to viruses that were likely integrated as proviruses at the time of sampling (S3 Table). However, these sequences could not be assigned to any MAG, likely due to their relative short length (average 2.5kb) and the presence of the viral sequences, which often display different k-mer frequencies and coverage values than the ones from their host genomes and distort the binning process. Additionally, functional prediction showed that only a small fraction of the sequences (6 out of 261 contigs) encoded integrase-like genes, indicating the capacity to integrate into the host genome. The only pleolipovirus-like element from the binned fraction (Gairdner_Node_15) was found integrated into the metagenome assembled genome of a new species belonging to the *Halobaculum* genus (S4 Table), for which no pleolipovirus has been assigned to the date. The pleolipovirus-like element of 10.852 bp is flanked by terminal repeats (TRs), with an integration site likely determined by the tryptophan tRNA adjacent to the integrase gene (S9 Fig), indicating that it is a complete genome.

***Frequent recombination and gene loss and gain of replication-related proteins, shape the structure of the Pleolipoviridae family***

Interestingly, the concatenated phylogenetic approach also provided some insights into the evolutionary history and acquisition of the replication machinery. The phylogenetic relationships uncovered in this work are consistent with the proposed scenario for the evolution of pleolipoviruses, with the last common ancestor of the *Pleolipoviridae* family likely acquiring the two variants of RCRE in independent events from different groups of plasmids (pGRB1-like pTP2-like plasmids, or a related ancestor). This event separated the members of this clade from the rest of the lineages giving birth to the *Alphapleolipovirus* genus [1,2]. Later in evolutionary history, the last common ancestor acquired the proposed replication protein of the *Aetapleolipoviruses* (Rep protein, corresponding to Halorubrum pleomorphic virus 3 ORF9) in an independent recombination event from a different group of plasmids or similar MGEs.

However, the phylogenetic reconstruction of the proposed Rep B protein in beta- and *Deltapleolipoviruses* displays a polyphyletic topology (S10 Fig), suggesting a more intricate evolutionary trajectory for these groups. Interestingly, the phylogenetic reconstruction of another conserved gene between the beta- and *Deltapleolipoviruses* groups (homolog proteins to ORF8 in HFPV-1), exhibit a clear separation between both clades (S11 Fig). This monophyletic characteristic shows that, despite being acquired at a similar time than the Rep B protein, it has remained relatively stable across the evolutionary history of both clades. The later suggests, that the Rep B replication protein was likely gained and/or lost multiple times in the evolutionary history. The later highlights once more that recombination events with other MGEs and between different pleolipovirus genera appear to be frequent in this groups of viruses. Furthermore it also suggests that the divergence of the beta and *Deltapleolipoviruses* likely occurred during earlier stages, at a similar time to the acquisition of the ORF8-like protein by the last common ancestor of the pleolipoviruses.

Altogether, while the replication apparatus in the *Alphapleolipovirus* genus remained relatively stable, the Rep protein was lost or replaced on multiple occasions, resulting in different lineages. We hypothesize that in the case of the *Gammapleolipoviruses* the Rep protein was replaced by a PolB type polymerase. Subsequently, different independent gene losses gave rise to the epsilonpleolipoviruses, which lack a canonical replication protein, or the proposed *Deltapleolipoviruses*, where some members do not exhibit any recognizable replication protein. The *Deltapleolipovirus* HFPV-1 for example, lacks a homolog to the conserved replication proteins and instead encodes a hypothetical protein with a Helix-turn-helix DNA binding domain, which has no homolog in any database [3]. Interestingly, while the *Alphapleolipoviruses* have a conserved replication strategy and a relatively conserved genome structure, the other lineages that went through a number of loss and gain events of the replication proteins have also in general a less stable core genome and are more prone to gene reshuffling and recombination with other MGEs and/or their hosts. The later suggests that the RCRE might be a more reliable enzyme for genome replication compared to the other uncharacterized proteins, which results in the observed higher genome stability. Under this scenario, given that the alternative mechanisms are more prone to make mistakes, the other clades may often rely on recombination as their main strategy for adaptation.

**Supplementary References**

1. Gorlas A, Krupovic M, Forterre P, Geslin C. Living side by side with a virus: Characterization of two novel plasmids from Thermococcus prieurii, a host for the spindle-shaped virus TPV1. Appl Environ Microbiol. 2013;79: 3822–3828. doi:10.1128/AEM.00525-13

2. Koonin E, Dolja V V., Krupovic M, Varsani A, Wolf YI, Yutin N, et al. Global Organization and Proposed Megataxonomy of the Virus World. Microbiol Mol Biol Rev. 2020;84. doi:10.1128/MMBR.00061-19CE

3. Alarcón-Schumacher T, Naor A, Gophna U, Erdmann S. Isolation of a virus causing a chronic infection in the archaeal model organism Haloferax volcanii reveals antiviral activities of a provirus. Proc Natl Acad Sci. 2022;119: 1–12. doi:https://doi.org/10.1073/pnas.2205037119
