# Supplementary figures and images for: Revisiting evolutionary trajectories and the organization of the Pleolipoviridae family
Source: PLoS Genet. 2023 Oct 13;19(10):e1010998. doi: 10.1371/journal.pgen.1010998 (PMC10599561; doi:10.1371/journal.pgen.1010998)

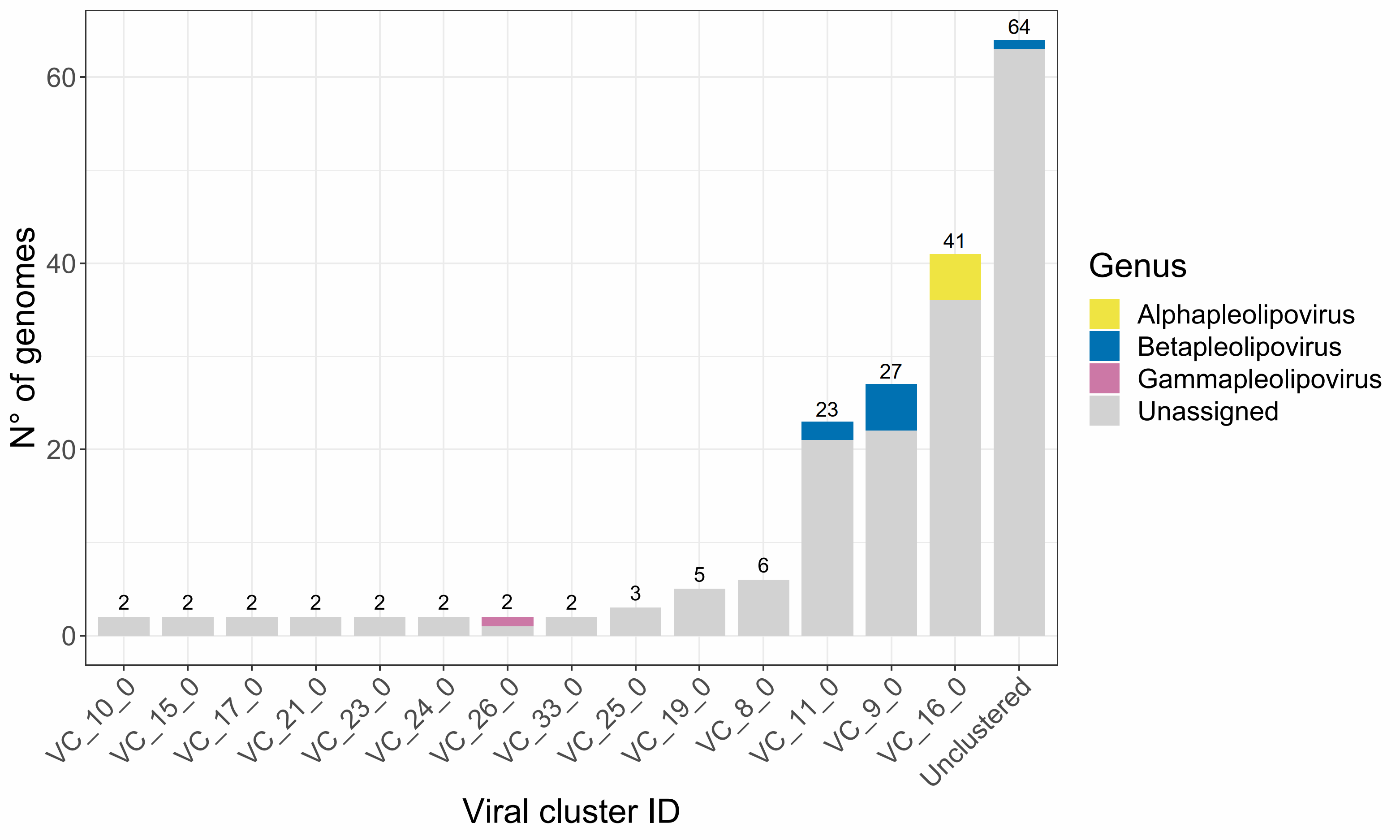

Supplement: S2 Fig — Pleolipovirus-like genomes were clustered based on their shared protein content with vConTACT2. Numbers on top of bars indicate the number of pleolipovirus genomes in the respective cluster. Colored labels represent isolated representatives from each genus: Alphapleolipovirus (yellow), Betapleolipovirus (blue), Gammapleolipovirus (magenta). Genomes classified as singletons, outliers and belonging to overlapping clusters were grouped under the category “Unclustered”. (TIF) [file pgen.1010998.s003.tif]

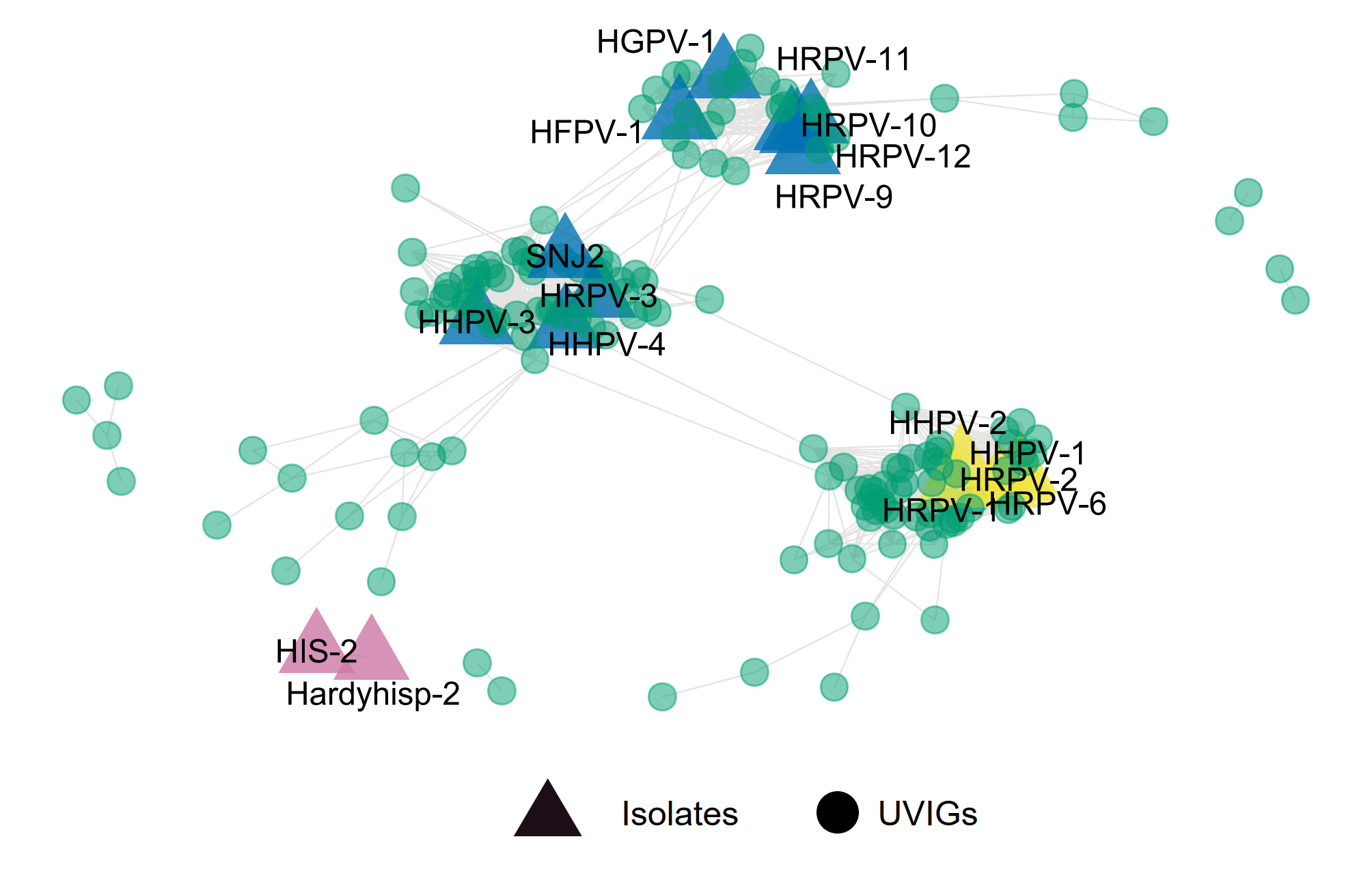

Supplement: S3 Fig — Nodes (circles) represent genomes and edges (lines) indicate shared protein content (minimum three protein clusters). Highlighted nodes are isolated representatives from the different genera: Alphapleolipoviruses (yellow), Betapleolipoviruses (blue) and Gammapleolipoviruses (magenta). (TIF) [file pgen.1010998.s004.tif]

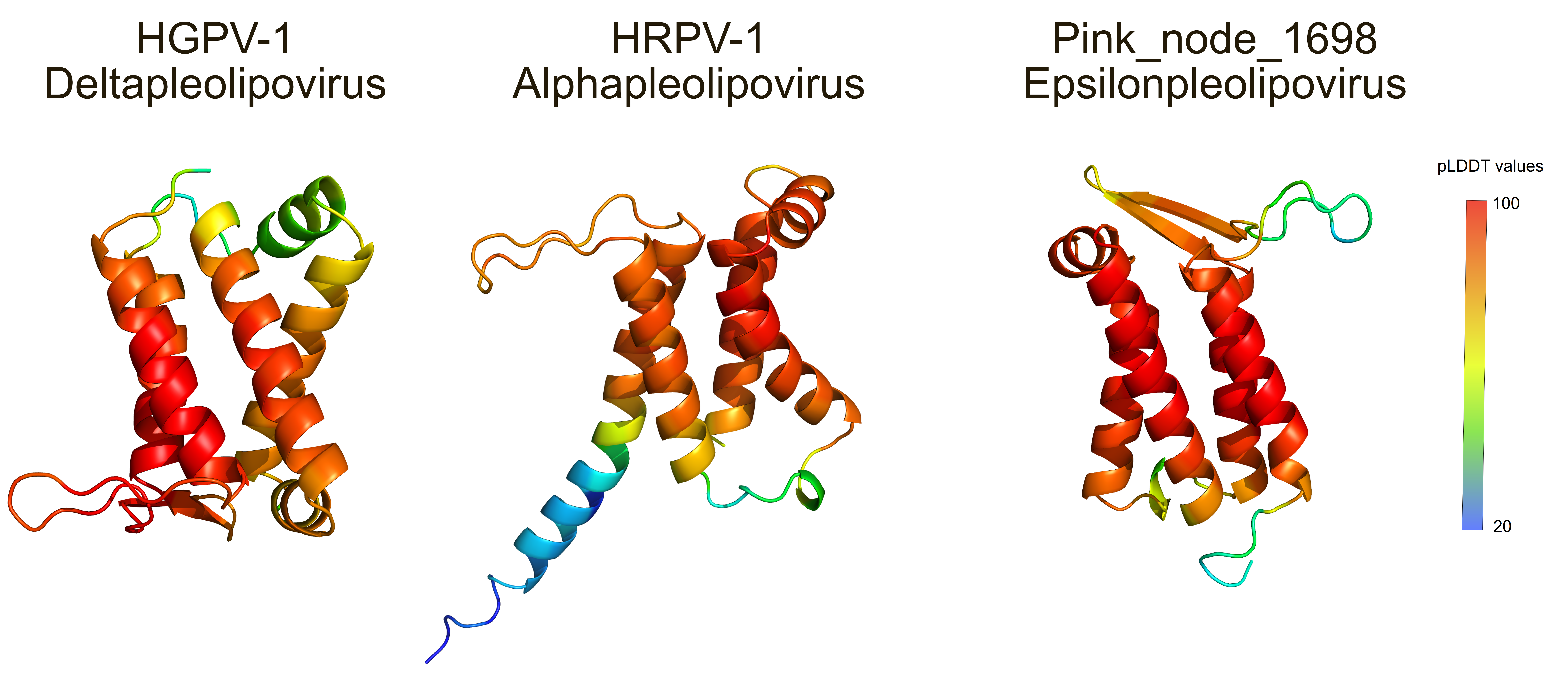

Supplement: S4 Fig — Structure prediction of the internal membrane protein type I (ORF2 in Haloferax pleomorphic virus 1) generated with AlphaFold2. Representative structures the genera Alphapleolipovirus (HRPV-1), Deltapleolipovirus (HGPV-1) and Epsilonpleolipovirus (Pink_Node_1698) are shown using ribbon representation and colored according to the plDDT values. (TIF) [file pgen.1010998.s005.tif]

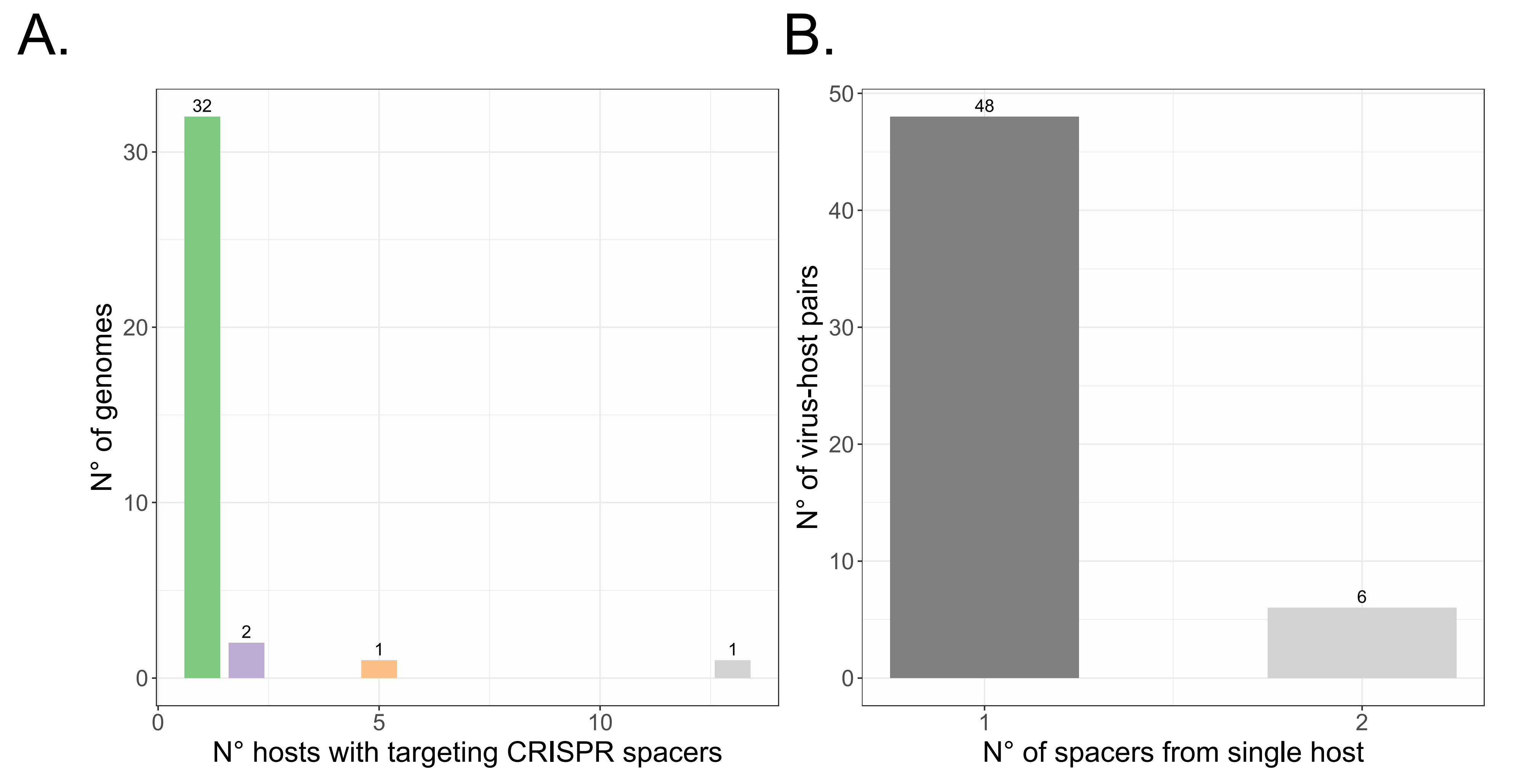

Supplement: S5 Fig — CRISPRs spacers were queried using blastn against the 184 pleolipovirus genomes from this study to assess the potential host range. A. Number of pleolipovirus genomes targeted by different hosts. B. Number of spacers from a specific host CRISPR array(s) for each one of the identified virus-host pairs. Numbers on top of the bars indicate the number of genomes and virus-host pairs respectively for A and B. (TIF) [file pgen.1010998.s006.tif]

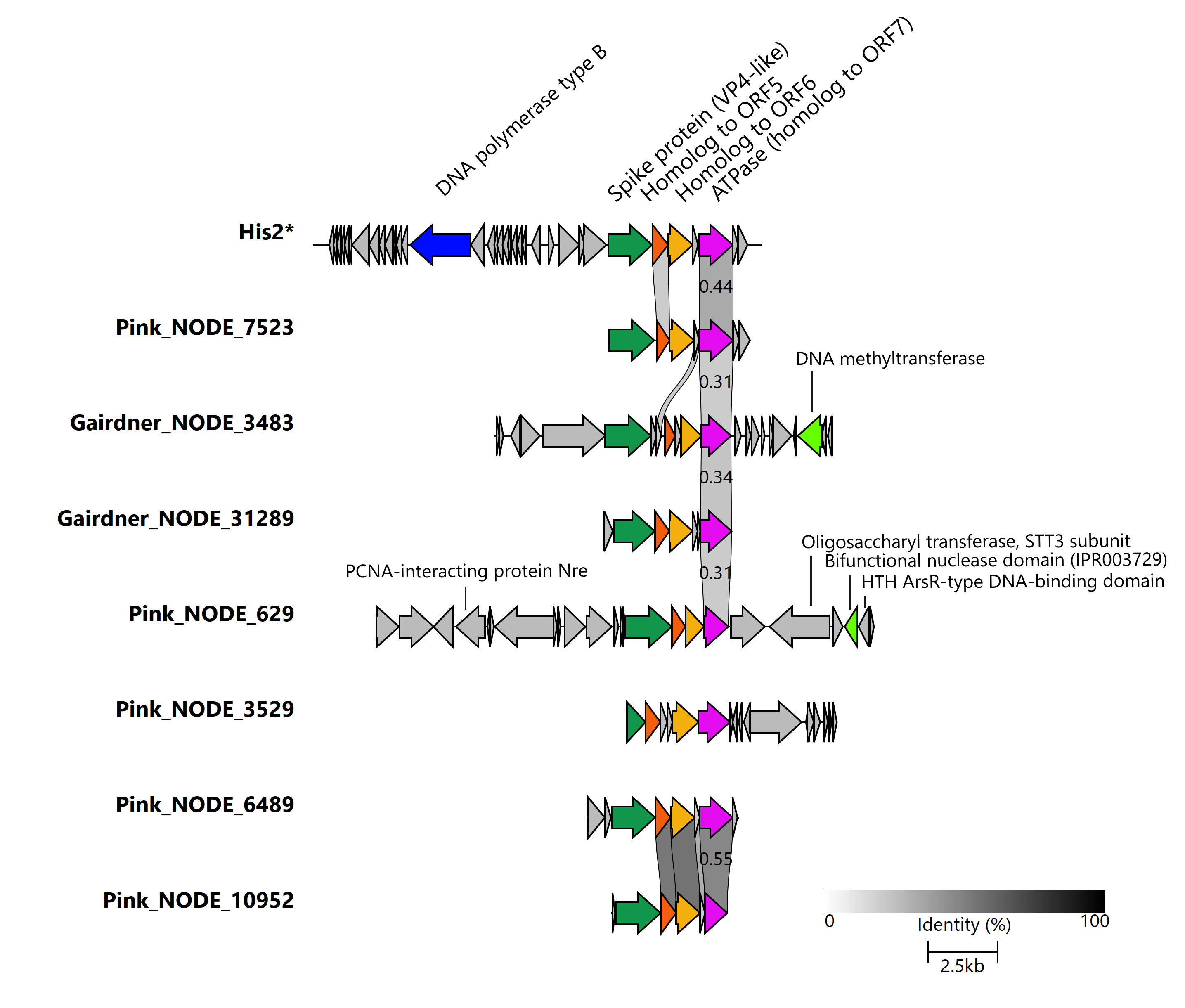

Supplement: S6 Fig — Genomic alignment of genomes related to the Gammapleolipovirus genus. Similarity values (blastp) are indicated by grayscale shading. Homologues of conserved genes are colored the same as follows: Spike protein (dark green), ORF5-like (red), ORF6-like (orange), ATPase (magenta), type B DNA polymerase (blue), and DNA methyl transferase (light green). Complete genomes are highlighted with (*) symbol. (TIF) [file pgen.1010998.s007.tif]

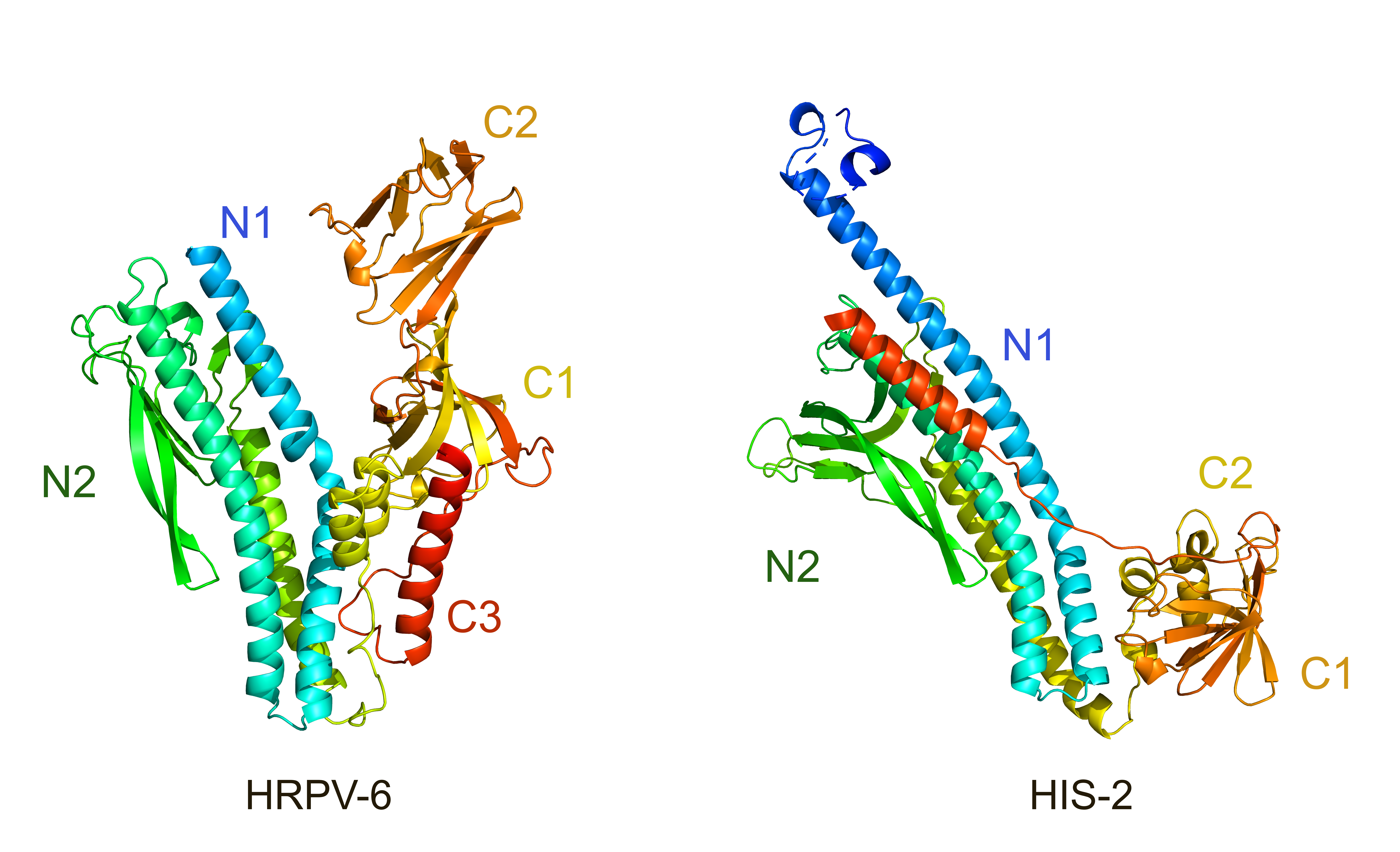

Supplement: S8 Fig — Structure prediction of the spike protein of the Alphapleolipovirus HRPV-6 and the Gammapleolipovirus His2 type I generated with AlphaFold2. Representative structures for major clades are shown using ribbon representation and colored using rainbow scheme from the N-terminus (blue) to the C-terminus (red). C1-C3 and N1-N2 indicate the protein subdomains. (TIF) [file pgen.1010998.s009.tif]

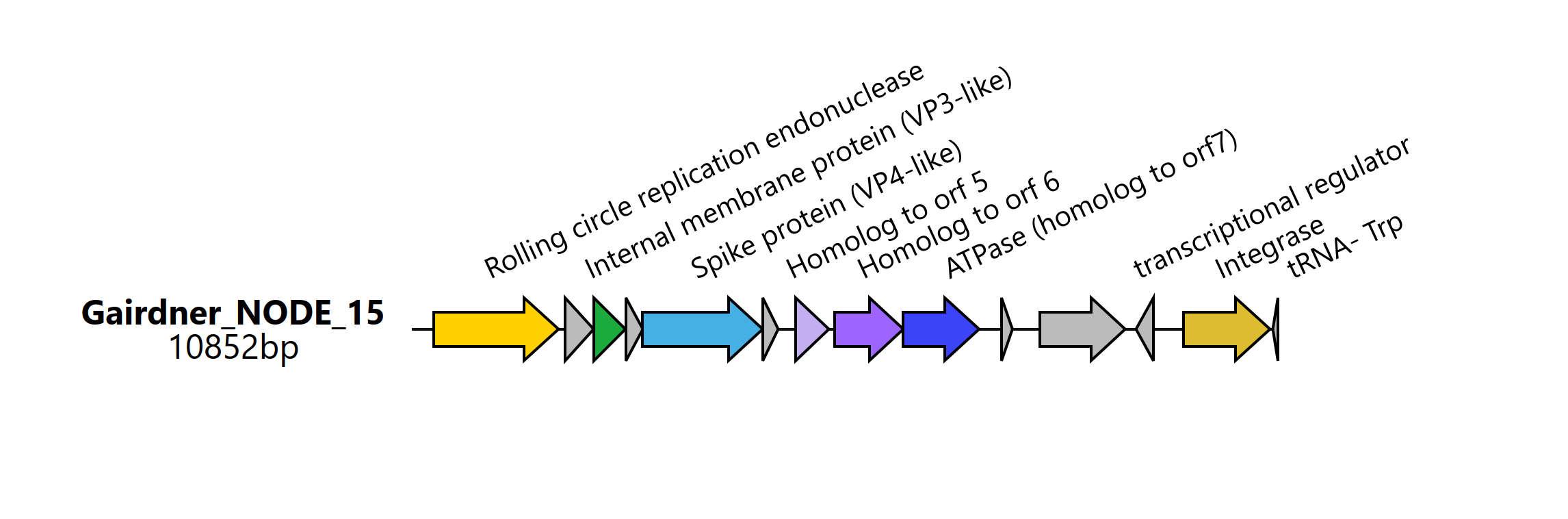

Supplement: S9 Fig — Genomic representation of newly identified Halobaculum pleolipovirus-like provirus. Homologues of conserved genes are colored the same as follows: Rolling circle replication endonuclease (RCRE) (yellow), VP3-like protein (green), Spike protein (light blue), ORF5-like (purple), ORF6-like (magenta), ATPase (blue) and integrase (light brown). Insertion site is depicted by the tRNA corresponding to tryptophan. (TIF) [file pgen.1010998.s010.tif]

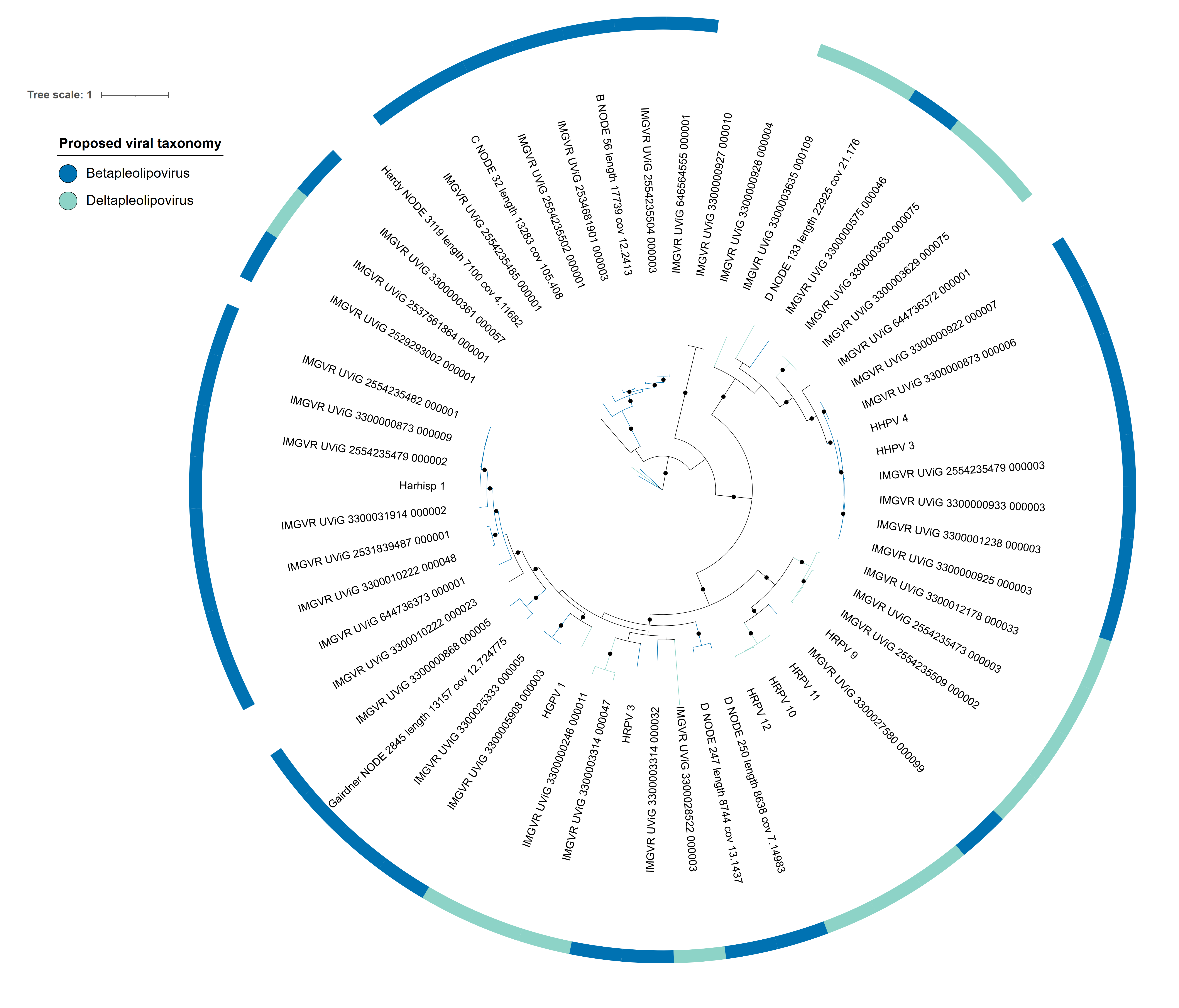

Supplement: S10 Fig — Phylogenetic tree reconstruction of the proposed replication-like proteins of Betapleolipoviruses. Sequences were obtained from the 184 pleolipovirus-like genomes dataset generated in this study. Tree was constructed with iqtree with 10.000 ultrafast bootstrap. Supported branches (SH-aLRT > = 80 and ultrafast bootstrap > = 95) are demarcated with black circles. Scale bar represents the number of substitutions every 100 amino acids. (TIF) [file pgen.1010998.s011.tif]

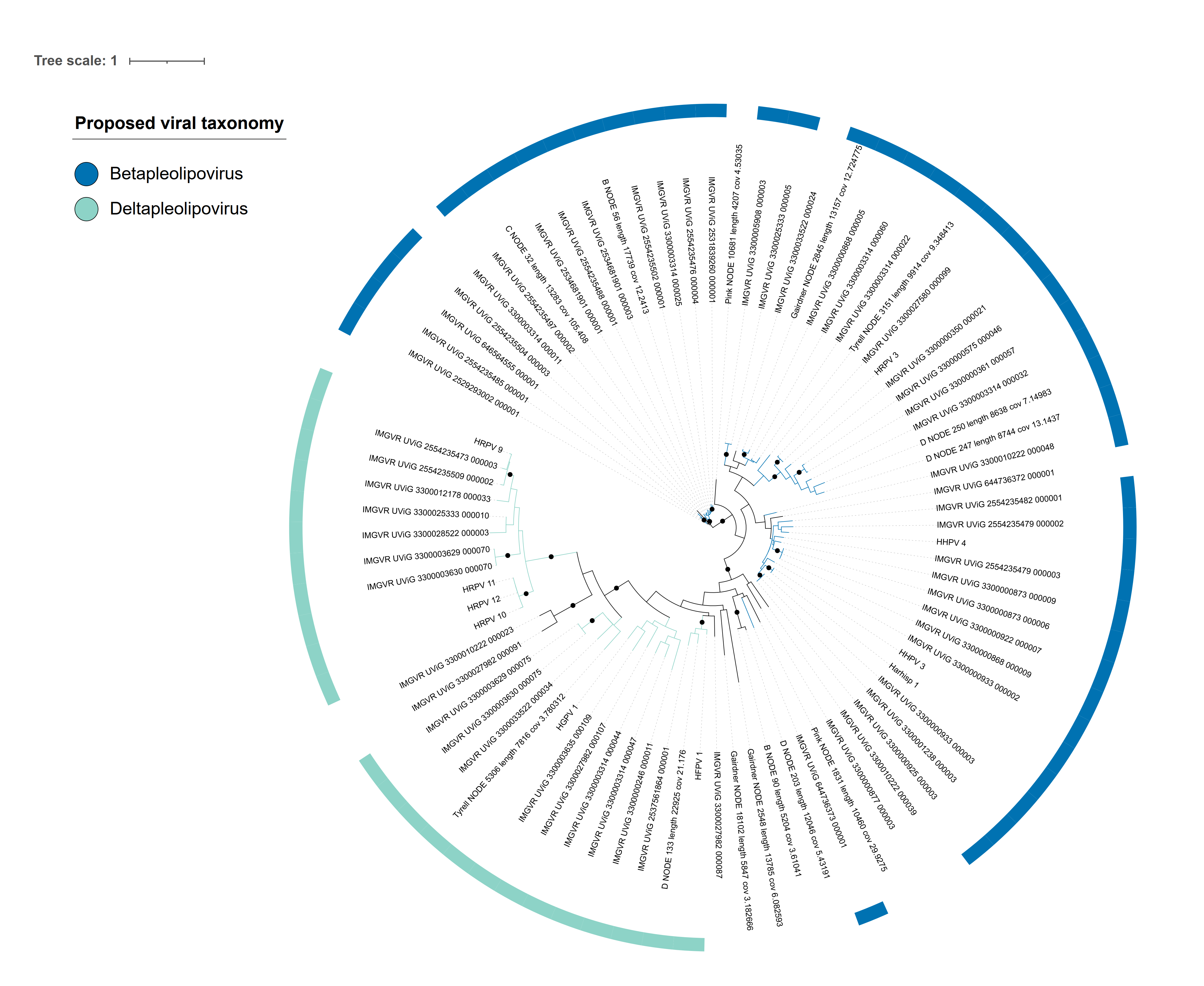

Supplement: S11 Fig — Phylogenetic tree reconstruction of ORF8-like proteins. Sequences were obtained from the 184 pleolipovirus-like genomes dataset generated in this study. ORF8 was designated according to the annotation of Haloferax pleomorphic virus 1 (HFPV-1). Tree was constructed with iqtree with 10.000 ultrafast bootstrap. Supported branches (SH-aLRT > = 80 and ultrafast bootstrap > = 95) are demarcated with black circles. Scale bar represents the number of substitutions every 100 amino acids. (TIF) [file pgen.1010998.s012.tif]
