# Supplementary material for: Revisiting evolutionary trajectories and the organization of the Pleolipoviridae family
Source: PLoS Genet. 2023 Oct 13;19(10):e1010998. doi: 10.1371/journal.pgen.1010998 (PMC10599561; doi:10.1371/journal.pgen.1010998)
Supplement: Attachment — Submitted filename: Response_to _reviewers.pdf [file pgen.1010998.s017.pdf]

POINT-BY-POINT RESPONSES TO THE EDITOR AND REVIEWER COMMENTS

We want to thank the editor and the reviewers for their time to critically evaluate our work and to point out weaknesses and errors within the manuscript. With your effort, the manuscript has improved substantially. We have considered all suggestions and concerns of the reviewers and addressed the main concern of Reviewer #1 by making sequence, annotation and additional files publicly available (Digital Object Identifier: 10.5281/zenodo.8248829).

Comments to the Authors:

Reviewer #1:

Pleolipoviruses represent one of the most widespread and diverse types of viruses infecting hyperhalophilic archaea. In this manuscript, Alarcón-Schumacher and colleagues explore the diversity of pleolipoviruses in metagenomic datasets generated from Australian hypersaline lakes as well as in publicly available databases, namely, IMG/VR. The authors report 164 new pleolipovirus genomes, greatly expanding our appreciation of pleolipovirus diversity and providing new insights into the evolution of this important virus group. The authors also use their data to shed light on certain aspects of virus-host interaction and suggest a revision to pleolipovirus classification. Overall, this is an important study, which following certain modifications outlined below, has the potential to be of great value to the community.

Response: Thank you for your positive feedback and for your suggestions.

For the results to be actually useful to others the authors have to either deposit the identified genomes to GenBank (as third party annotations in the case IMG/VR contigs and as new entries for those assembled from the new data) or, at the very least, provide all the genomes as GenBank and fasta formatted files in the supplementary information. Currently, the authors only deposited the raw reads, which is good, but not sufficient. If I or someone else were to reassemble the reads, we would obtain a different set of contigs and would have no way of comparing with those reported in this manuscript. Also note that even if the contigs can be downloaded from IMG/VR, they are not annotated and gene calling could produce different results in other studies. Thus, please provide all the data in one place so that others could build upon it rather than regenerating it from scratch.

Response: The data analyzed in this study were made available and are readily accessible in an online repository (Digital Object Identifier: 10.5281/zenodo.8248829). The data includes individual assembled virus genomes and contigs used in this study (PV_fasta.tar.gz), protein prediction (PV_fna.tar.gz and PV_faa.tar.gz for nucleotide and amino acid sequence of gene prediction respectively), along with protein annotation (PV_proteins_eggnogmapper.tab), HMM models generated for specific genes (Hmm_models.7z) and structural predictions of spike protein with Alphafold2 (PV_alphafold2_models_spike_protein.tar.gz).

The manuscript is too long and could be shortened by removing some generic speculations or moving some sections to the supplement (intended for the biggest pleolipovirus enthusiasts). For instance, "Frequent recombination and gene loss…" is one of such sections, as it presents little new insights and is repetitive with what was said before in the manuscript.

Response: Thank you for this suggestions. We moved the suggested section to Supplements and shortened the remaining manuscript where possible.

It is not clear why the authors chose not to trim the alignments (line 168), contrary to the widely accepted standards in phylogenetics. This decision might have affected the results and is particularly pertinent to the claims by the authors that VP4 is a bad phylogenetic marker. The impact of trimming is expected to have different impact for short and long proteins, hence, the lack of congruence in phylogenies produced for different proteins. Phylogenetic analyses have to be repeated with properly trimmed alignments to exclude this possibility.

Response: Phylogenetic analysis were performed both with and without alignment trimming. We tested two different trimming softwares independently: Trimal (https://doi:10.1093/bioinformatics/btp348) and ClipKIT (https://doi.org/10.1371/journal.pbio.3001007) with almost identical results. The observed lack of congruence reported before for ORFs 4 (spike protein) and 6 (unknown function) was consistent regardless of the approach used. We presented the results from untrimmed sequences in the previous version of the manuscript, because it still debated in the literature whether some trimming strategies can introduce a bias (https://doi.org/10.1093/sysbio/syv033). Nevertheless, we include now in the main manuscript and figures the versions of the trees were trimming of the alignments was performed (Figure 3 and 4) and specified the strategy used in the methods section.

L206-230: The presented data does not quite convince me that "Novel pleolipovirus-like elements from Australian salt lakes reveal the preference for a productive life cycle". Most of the contigs are very short (cutoff of 2 kb), which might not be sufficient to make assessment of integrated vs extracellular state.

Response: We have removed this section. We agree that more evidence is required to sustain such a claim.

224-230: This part is overly speculative and obscure given that impact of pleolipoviruses on host expression has been shown only for one virus, with all other isolates being apparently rather benign.

Response: We have removed this paragraph. We agree that more evidence from other pleolipoviruses and their impact in metabolic remodeling is required to sustain these claims.

L279: If I am not mistaken, vContact genus-level clusters are identified when 3 genes are shared. The authors settled for 2 shared genes. If so, the genus-level calibration might be off.

Response: You are correct. We have intentionally modified the threshold for the network analysis in vContact. The pleolipovirus genomes show very low levels of similarity at the amino acid sequence level, even for homolog proteins the sequence identity is often below the thresholds used for blast analysis. Additionally, pleolipoviruses exhibit on average much smaller genomes than bacteriophages for which the software was initially designed. These factors tend to artificially weaken the signal and thus the product is a large number of small genus-level clusters. By using the 3 gene threshold many of the connections present in the 2 gene analysis are lost, in particular between the more divergent Pleolipovirus genomes e.g. the *Gammapleolipovirus* (see new Supplementary figure 3). Nonetheless, the overall patterns of the network remain unchanged.

For the proposed taxonomy, we do not only use the vContact analysis as single criteria, but rather the overall results from the different approaches. Thus, we would not recommend to the split the family in a myriad of genera without stronger biological evidence to support it. Naturally, we cannot exclude that with the discovery of additional representatives of the family, the taxonomy will need further revision, but we believe the current evidence strongly supports our proposal. We now included the analysis using the 3 gene threshold into the supplements and discuss the results accordingly.

L290-291: I am really not fond of this sentence: "Altogether, this indicates that the diversity within the Pleolipoviridae family could be greater than previously thought and further challenges the current classification." It is obvious that diversity within any given virus family is greater than currently sampled and classification has to be constantly revised and expanded once new viruses are discovered. Same comment for statement on L346 – "greater than previously thought" – thought by whom? I personally think that that even after this study we are still very far from appreciating the true genomic diversity of these viruses.

Response: We have considered the reviewer suggestion and the statements have been removed from the text.

L293: "Betasphaerolipovirus" genus does not exist and the family no longer includes Thermus phages (PMID: 37093734).

Response: Thank you for pointing this out. It has been corrected in the text according to the reviewer suggestion.

L321: Throughout the manuscript the authors keep referring to core genes, referring to HFPV1. First, show a genome map in one of the first figures (upon first mention of core genes). Second, in all other papers on pleolipoviruses HRPV-1 is used as a reference. Given that the latter virus has been studied much more extensively than HFPV1, it does not appear justified to readopt the gene nomenclature – this just brings confusion.

Response: Thank you for pointing this out. We revised the annotation throughout the text and refer to HRPV-1 ORFs as suggested by the reviewer. With the exception of proteins lacking a homolog in HRPV-1 (ORF8 like in HFPV-1 and RepBeta-like proteins). Therefore, we believe it is now enough to refer to the published genome map in https://doi.org/10.1073/pnas.2205037119 without including an additional genome map of HFPV-1 into the manuscript.

L328 (again on L401): There is no such thing as "very polyphyletic nature" – it is either polyphyletic or not.

Response: Thank you for pointing this out. It has been corrected in the text according to the reviewer suggestion.

L357: I am not convinced that this genome is complete. ITR would strongly suggest the presence of a protein-primed DNA polymerase, like in His2, and there does not seem to be one in this virus. Could the inverted repeats actually represent inverted repeated genes?

Response: You are correct, while both VirSorter2 and CheckV suggest this is an ITR, this could be indeed a small inverted ORF. We have manually revised the automated protein prediction, annotated the

repeated ORF in question (-57aa protein- with no function predicted), and updated the protein prediction file (available in the online additional material). Accordingly, we have designated the other complete genomes from the proposed *Epsilonpleolipovirus* as representatives and stated so in the text.

L380-383: The choice of examples is rather strange. Why His2, which encodes its own polymerase?

Response: Thank you for pointing this out. The examples have been revised and rewritten to avoid misunderstandings.

L384: I would suggest refraining from linking HNH to terminase. HNH nucleases perform so many different roles, which are more likely than the one offered.

Response: We agree that the actual function needs to be elucidated in order to sustain such a claim. Therefore, we removed the sentence to avoid misleading the reader.

The Abstract is overly long and tedious. Reads more like an Introduction. Please consider shortening.

Response: Thanks for the suggestion, we have shortened he abstract according to the reviewers' suggestion.

All virus taxa should be written in Italics (e.g., line 32).

Response: This has been revised throughout the text.

L40: "one new genera" > "… genus".

Response: This has been corrected in the text. (Line 36)

L41: The authors do not demonstrate that genome size is dependent on the ability to integrate. No causality is demonstrated. There might be correlation (should be statistically significant), but not more than that.

Response: There are statistically significant differences in the genomes size between those genomes carrying integrases and those which do not, regardless if considering all genomes or only those deemed complete (p-value =2.2×10^{-16}, see *Network analysis reveals a correlation between genome length and integration section*). However, we agree with the reviewer that no mechanistic evidence is presented and we have rephrased the sentence to avoid misleading the reader.

L47: "analysis of virion spike proteins reveals that the evolution of the spike protein is driven by the interaction with the cellular surface of the hosts" – this sounds trivial and no data is presented to make a more concrete statement.

Response: It has been rephrased and clarified in the text.

L88: "the only isolated member of the Gammapleolipovirus genus" – why was Hardyhisp2 excluded (PMID: 33986080)? This virus has been isolated, not merely sequenced.

Response: Thanks for pointing this out. We acknowledged that Hardyhisp2 has also been isolated and this has now been corrected in the text.

Please mention that numerous proviruses related to alpa and betapleolipoviruses have been described previously (e.g PMID: 29361162 etc).

Response: We have acknowledged these now in the text and added the corresponding reference to the text.

Reviewer #2:

Archaea viruses are highly diverse, and with the recent changes to ICTV phage taxonomy classification guidelines, much work needs to be done to reorganize viral taxonomy. The authors identify 129 high-confidence genomes through metagenomic sequencing belonging to the Pleolipoviridae family. They then use this data to propose two new viral genera within the Pleolipoviridae family and predict the origins of these chronic infecting phages.

The paper is very well written with a straightforward story. The authors provide multiple pieces of evidence showing that the Pleolipoviridae phage family needs to be further broken down into additional phage genera using phylogenetic trees, whole genome comparisons, and vConTACT2. It also dives into the evolutionary history of pleolipoviruses and the impact of recombination on the diversity of the viral family. The figures, especially the phylogenetic trees, are very blurry, and parts of the tree are impossible to see. Much work needs to be done on improving figure quality though it could be an issue when uploading the document.

Response: Thank you for your comments. We have considered all suggestions and concerns and addressed them in the revised text. Regarding the quality of the figures, all figures files were provided as high-quality images (minimum 400 dpi) upon submission. Likely the loss of quality is due to the submission system. We now include the figures into the manuscript file to prevent the loss of resolution.

Minor comments

Line 32 Pleolipoviridae in the abstract needs italicized.

Response: Thank you for pointing this out. It has been corrected in the text.

Line 34 The sentence has two very different concepts: infection mechanisms and how they evolve. Delete "and" and start a second sentence beginning at interestingly.

Response: This sentence has been removed from the abstract.

Line 85 italicize Betapleolipovirus since that is a genus name.

Response: Thank you for pointing this out. It has been revised throughout the text.

Line 191, there is a parenthesis "(ORFs… that does not connect to anything. I would recommend deleting that.

Response: It has been clarified in the text.

Lines 191-200 A lot of what is mentioned is already mentioned in the methods section and could be further summarized.

Response: This section has been shortened and combined with the following section according to both reviewers' suggestion.

Line 205. Mentioned 129 genomes were selected for further genome comparisons. There should be a table with all of the genomes with accession numbers referenced.

Response: Selected genomes are summarized in supplementary Table 1, along with their respective identifier from the IMGVR database. Unfortunately, these do not have a reference in the NCBI database, but we have now made all sequence and additional files available for download (Digital Object Identifier: 10.5281/zenodo.8248829).

Line 206, the title is slightly ambiguous. How do the following paragraphs reveal the preference for a productive life cycle? Do you mean a productive, persistent life cycle?

Response: This section has been revised and the heading of this section has been modified accordingly to better convey the message.

Line 440. The authors mention that the viruses have a narrow host range, but it would be useful to define what they consider is a novel host range, i.e., strain, species, genera, and family-level host ranges. You mention in the following sentence that HFPV-1 infects multiple families, so it is confusing how narrow of a host range the majority of other phages are.

Response: We have revised the phrasing and further clarify what is considered a narrow v/s broad host range.

Major comments

In the abstract, you say you identified 164 new genomes, but in the text, your search yielded 184 non-redundant pleolipovirus genomes (line 246), and 129 of those were used for phylogenetic analysis (line 204). How many novel viruses did you discover, and where does 164 come from?

Response: The 184 non-redundant genomes include the genomes from the isolates (17 isolates) and three genomes reported before from uncultivated viral genomes in a previous publication (DOI: 10.3390/genes12020149). Hence, there are 164 novel curated genomes. From these 164, 41 come from the metagenomic data produced in this study (Australian hypersaline lakes) and 126 from *de novo* annotation and curation of the IMG/VR database and publicly available datasets. The origin of each viral genome is specified in the Supplementary table 1. This has also now been specified in more detail in the abstract and in the main text.

Figure 2 is very blurry. I am unsure if it is just due to how the picture was uploaded, but I cannot read the conserved features keys, and the entire tree is blurry. The conserved feature key should have the color and shape of the key. It would also be helpful if the internal membrane protein I was not square. When first glancing at Figure A, it looks like the outside ring is showing that the phages belong to alphaplelipovirus.

Response: The figure has been revised according to the suggestions of the reviewer. Regarding the quality, all figures were provided as high-quality images (minimum 400 dpi) and likely the loss of quality is due to a system issue non-related to the authors. We now include the figures into the manuscript file to prevent the loss of resolution.

To establish a new viral taxon, you must have complete viral sequences in the proposed viral genera. It would be useful to add in the text or figures showing the phages with complete genomes. You can refer to this paper for the most recent guidelines for uncultivated virus genome taxonomy classification https://www.nature.com/articles/s41587-023-01844-2 (Adriaenssens et al., Nature Biotechnology, 2023)

Response: The reviewer is right. We have followed the guidelines proposed by Addriaenssens et al, and the data on genome completeness is provided in supplementary table 1. We added a summary for each proposed genus in the text now.

It may be worth proposing a subfamily for the beta, gamma, and epsilonviridae phages since the majority do share the rep protein and have circular double-stranded DNA genomes with single-stranded discontinuities.

Based on vConTACT2 and ANI, there appear to be many more genus clusters within the Pleolipovirus-like genomes. It may be worth mentioning that although you may not have enough data to support additional genera, some viruses remain unclassified.

Response: Regarding proposing a subfamily for the aforementioned clades, we believe that in order to do so, more biological data is required, ideally in the form of additional isolates from these novel clades, to for example determine the nature of their nucleic acid. Furthermore, the fact that the proposed replication protein does not provides a clear phylogenetic signal (which could be a product of multiple gain and loss events) further refrain us to do so. Nonetheless, we agree that vContact clustering suggest that there might be a need to add new genera as our knowledge on the diversity increases. However, it is not our intent to propose multiple small genera with one or two representatives, which would further complicate the classification. Instead we aim to provide a reasonable structure of the family for further studies of pleolipoviruses. We describe this now in more detail in the text.
